# Epidemiology of Phenylketonuria Disease in Jordan: Medical and Nutritional Challenges

**DOI:** 10.3390/children9030402

**Published:** 2022-03-11

**Authors:** Safwan Dababneh, Mohammed Alsbou, Nashat Taani, Ghazi Sharkas, Refqi Ismael, Latifeh Maraqa, Omar Nemri, Hanin Al-Jawaldeh, Nadeen Kopti, Enas Atieh, Arab Almasri

**Affiliations:** 1 Ministry of Health, Amman 11118, Jordan; safwanjo@yahoo.com (S.D.); nashaattaani@yahoo.com (N.T.); ghazisharkas@yahoo.com (G.S.); august_mahmoud@yahoo.com (R.I.); latifa_maraqa@hotmail.com (L.M.); onimri@gmail.com (O.N.); haljawaldeh@yahoo.ca (H.A.-J.); nadeen.kopti@hotmail.com (N.K.); enas.ateia@yahoo.com (E.A.); arab.abed79@gmail.com (A.A.); 2 Faculty of Medicine, Mutah University, Karak 61710, Jordan

**Keywords:** phenylketonuria (PKU), national newborn screening (NBS) program, dietary management, Jordan

## Abstract

Background: Phenylketonuria (PKU) is the most frequent inborn error in amino acid metabolism caused by a deficiency of the phenylalanine hydroxylase enzyme (PAH). If PKU is left untreated, high concentrations of phenylalanine (Phe) accumulate in the blood, leading to severe brain dysfunction, neurodevelopmental, behavioral and psychological problems. Data concerning the epidemiology of PKU in Jordan are limited. The main objectives of our study were to determine the prevalence of PKU in Jordan, analyze the PKU phenotypes, and identify major challenges in providing dietary management to PKU patients. Methods: Data were collected utilizing the medical records of PKU patients attending the PKU clinic at the Ministry of Health in Amman, Jordan, between 2008 and 2021. Results: The total number of patients diagnosed with PKU was 294. The prevalence of PKU was estimated to be 1/5263. Most patients were Jordanians (90.8%), and 9.2% were non-Jordanians. More than half of the patients (56%) were diagnosed through the national newborn screening (NBS) program. Regarding the phenotypes of PKU, 46.6% had moderate PKU, whereas 42.9% had the classic type of PKU and only 8 (2.7%) had cofactor Tetrahydrobiopterin (BH4) deficiency (atypical PKU). According to the age of diagnosis, 66% of patients were diagnosed more than 30 days post-birth. Consanguinity was found in 87.4% of patients, and the majority of patients, 218 (74.2%), had first-degree consanguinity. The most common complication was mental retardation (31%). Most patients were committed to dietary management (83%) and developed fewer complications. Conclusion: In our study, we demonstrated the importance of the NBS program in the early identification and diagnosis of new PKU cases which allows the initiation of treatment and dietary management to prevent severe complications of PKU in Jordan.

## 1. Introduction

Phenylketonuria (PKU) (OMIM 261600) is an autosomal recessive disorder caused by a deficiency of phenylalanine hydroxylase (PAH). PAH catalyzes the hydroxylation of phenylalanine to tyrosine using tetrahydrobiopterin (BH4) as a cofactor [1]. PKU is the most common inborn error of metabolic disorders and was first described in 1934 by the Norwegian physician Asbjorn Folling [2]. There are four types of PKU phenotypes based on the phenylalanine (Phe) concentration in plasma: mild hyperphenylalaninemia (120–600 μmol/L), mild PKU (mPKU; 600–1200 μmol/L), moderate PKU (900–1200 μmol/L), and classic PKU (cPKU; >1200 μmol/L) [3]. If PKU is not detected during the first week after birth or left untreated, Phe will accumulate in the blood and brain, leading to severe intellectual disability, psychological and behavioral problems, epilepsy, eczema, and light pigmentation of the hair and skin [4]. Therefore, the early identification of cases and early treatment are essential to preventing the complications of PKU.

The national newborn screening (NBS) programs for PKU have been remarkably successful. NBS for PKU is performed by the collecting of a blood sample from the heel prick of all newborns on a filter paper card. The collection of blood samples is completed between 24 and 72 h after birth. Blood specimens are then sent to a public health laboratory for further biochemical analysis. If the result is abnormal, laboratory staff notify the PKU clinic to follow up the case. Newborn screening is usually completed by using tandem spectrometry (MS/MS) [4]. When diagnosed early in the newborn period and treated to achieve good metabolic control, people can proceed to have a normal, healthy development and live lives of average length. The first NBS program for PKU was implemented in the USA in 1960, followed in several other countries during the next few years [5,6,7,8,9,10]. The first PKU screening project in Jordan was initiated in collaboration with the Ministry of Health (MOH) in 2006. The MOH in Jordan began the NBS program for PKU, congenital hypothyroidism (CH) in 2008, and Favism (G6PD-deficiency) in 2012 for all newborns, including Syrians and refugees from other countries [11]. Furthermore, we have only one specialized clinic in Jordan to monitor and treat PKU patients. The clinic manages individuals with PKU from diagnosis through newborn screening and into adulthood. We have a team approach in providing care to our patients. The PKU clinic team includes two pediatricians, a nurse, and three volunteer dietitians. Since the main PKU treatment is a lifelong low-protein diet, the MOH distributes only phenyl-free milk formula and low-protein flour free of charge to all age groups and nationalities throughout their lives. Nutritional monitoring includes recording anthropometric measures (weight, height, and head circumference) to ensure normal growth. We also perform the following at each visit: food recalls, the calculation of daily energy needs (daily calories), the calculation of daily phenyl-free protein and natural protein requirements, the provision of alternative low-phenyl recipes and meal plans and providing solutions to render phenyl-free milk more palatable. We aim to promote self-care by providing nutritional counseling and education to parents and families, particularly by teaching them how to calculate the phenylalanine content in different food products.

A Phe-restricted diet has been the mainstay treatment for PKU. Children treated early for PKU demonstrate subtle problems in cognitive function, school achievement, behavioral adjustment, and quality of life. The current treatment for this disorder involves strict metabolic control using a low-Phe diet of specialized medical foods. Small amounts of Phe from breast milk or commercial infant formula are considered sufficient for babies. In older children, the daily protein requirement is calculated, whereby a child is allocated a certain number of grams or units of daily protein depending on longitudinal plasma Phe concentrations. Foods such as eggs, milk, cheese, meat, poultry, fish, dried beans, and legumes, which are high in protein, are excluded from the diet [12]. The treatment of neonates born with PKU should be initiated as soon as possible, no later than seven days after birth. Phe levels should be controlled immediately, and breastfeeding is encouraged along with a Phe-free formula [13].

The PKU prevalence and incidence differ between different ethnic groups and geographic regions. The overall incidence in Europe is 1 in 10,000 newborns, in Ireland 1 in 4500, and in Turkey 1 in 2600 [14,15]. The prevalence of PKU in Jordan is not known yet. To our knowledge, this is the first study attempting to collect data on the prevalence, diagnosis, and dietary management of PKU in Jordan. The main objectives of our study were to determine the prevalence of PKU in Jordan; the phenotypes, gender, and geographic distribution of PKU cases; the relationship between consanguinity marriages and affected patients; the dietary challenges for PKU patients; and finally, the role of the NBS program in detecting PKU among newborns in Jordan.

## 2. Materials and Methods

We conducted a retrospective descriptive study. Data were collected from the records of all PKU patients attending the MOH’s PKU clinic from 2008 to 2021. All PKU patients in Jordan must attend this clinic to receive their free dietary formulas and a follow-up with qualified staff and metabolic dietitians. The following data were recorded: age, gender, age at diagnosis, consanguinity of parents, complications, developmental milestones, follow-up of dietary treatment, PKU phenotypes, area of residence, and nationality.

### Statistical Analysis

The collected data were filtered, and coded according to the required needs and specifications, and it was stored in an Excel format with a protection arrangement. Variables are presented as numbers and percentages. A *p*-value less than 0.05 was considered significant. The data were entered and analyzed using the Epi Info statistical program version 7.2.

## 3. Results

The total number of patients attending the PKU clinic during the study period was 294. The prevalence of PKU in Jordan is estimated to be 1/5263. Regarding sex distribution, more than half of the patients were males (269, 57.5%), and 125 (42.5%) were females. A total of 267 patients were Jordanians (90.8%), and 27 were non-Jordanians (9.2%). Regarding the age of diagnosis, 194 patients (66%) were diagnosed more than 30 days post-birth, and 28 patients (9.5%) were diagnosed during the first week of life. More than half of the patients (165, 56%) were diagnosed through the newborn screening program for PKU. Regarding the PKU phenotype of PKU, 137 patients (46.6%) had moderate PKU, followed by 126 (42.9%) who had the classic type of PKU, and only 8 (2.7%) had the variant BH4. Consanguinity was found in 257 (87.4%) patients. A total of 218 patients (74.2%) had first-degree consanguinity, and 29 (9.9%) had second-degree consanguinity. Only 37 patients (12.6%) were not consanguineous. The most common complication was mental retardation (91, 31%); 16 patients (5.4%) suffered from epilepsy; 10 (3.4%) had attention deficit hyperactivity disorder (ADHD); and 55.4% of patients had no complications. Regarding developmental milestones, 169 patients (57.5%) had no developmental delays compared with 125 patients (42.5%) who had developmental delays. Regarding the geographic distribution of PKU patients, 47.62% lived in Amman, followed by Zarqa at 16.33%, and Irbid at 10.54% (Table 1).

Regarding the dietary management follow-up, 83% of patients (244) were committed to dietary treatment and had fewer complications (mental retardation, ADHD, cerebral palsy, epilepsy, osteopenia) compared with 17% of patients (50) who were not committed and developed more complications (Table 2).

Since 2008, when the NBS program was established in Jordan, 171 PKU patients (58%) born after 2008 were diagnosed compared with 120 patients (41%) born before 2008. More than half of the patients (69, 58%) born before 2008 had mental retardation compared with only 21 patients (12%) born after 2008. Two-thirds of patients (86, 72%) born before 2008 had developmental delays compared with 37 patients (22%) born after 2008. A total of 162 patients (95%) born after 2008 followed a special PKU diet compared with 78 patients (65%) born before 2008. These results indicate the importance of the NBS in the early identification and diagnosis of new cases, as well as providing proper dietary management to prevent PKU complications (Table 3).

As for the age of diagnosis, patients diagnosed more than 30 days post-birth (late-diagnosed patients), between 8 and 30 days post-birth, and in the first week post-birth experienced complications at rates of 59%, 11%, and 7%, respectively. Patients diagnosed more than 30 days post-birth had a higher incidence of complications. About half of the patients (88, 45%) diagnosed more than 30 days after birth had mental retardation compared with two patients (2.8%) diagnosed between 8 and 30 days after birth. Only one patient (3.6%) was diagnosed in the first week outside the womb. More than half of the patients (117, 60%) diagnosed more than 30 days post-birth had developmental delays, whereas 6 patients (8%) diagnosed between 8 and 30 days post-birth had developmental delays. Only two patients (7%) were diagnosed in the first week post-birth. These results indicate that the early identification and diagnosis of patients is important in treating and preventing dramatic complications from PKU (Table 4).

## 4. Discussion

The prevalence of PKU differs significantly between different ethnic groups and countries worldwide (1:4500 in Italy, 1:125,000 in Japan) [16]. There is little data about the prevalence and incidence of PKU in the Middle East. A systemic review investigated the prevalence of PKU in Arabic countries and reported high rates in Saudi Arabia, the United Arab Emirates, Gaza Strip, and Iraq [8]. Information about the prevalence of PKU is lacking in Jordan. Our study demonstrated a high prevalence of PKU in Jordan (1 in 5263). The high rate of consanguineous marriages may explain this high prevalence. Furthermore, our results indicated a high degree of consanguineous marriages (87.4%) and mainly first-degree consanguinity (74.2%). Another study investigating the spectrum of inborn error of metabolic diseases in Jordan found that 137 out of 151 families had consanguineous parents [17]. These results are consistent with previous studies from other Arabic countries. A high degree of consanguinity has been reported in other Arabic countries: 8 out of 9 PKU patients in Kuwait and 9 out of 11 in Oman had parental consanguinity [18,19].

The NBS program aims to screen for congenital and heritable disorders. If treated, people may have relatively normal lives with reduced medical costs over time; however, if left untreated, these disorders can result in severe mental retardation, growth problems, developmental delays, behavioral/emotional problems, deafness or blindness, seizures, coma, and occasionally death [20,21]. According to our results, more than half of the PKU patients were detected through the NBS program (56%). Other cases were diagnosed by selective screening of affected families with PKU. In Jordan, and due to improvements in nutrition and the control of non-communicable diseases, genetic disorders have become a concern for the public and healthcare providers; however, Jordan has a limited NBS program that only targets three metabolic diseases: PKU, congenital hypothyroidism (CH), and favism (G6PD-deficiency). The high rate of consanguineous marriages in Jordan has increased autosomal recessive conditions, including inborn errors in metabolism. Therefore, a comprehensive newborn screening service must be introduced to the Jordanian community to diagnose such disorders early and to provide appropriate follow-up management and treatment [17]. A recent review investigated the prevalence of PKU in Arab countries with an NBS, including Turkey and Iran. The results showed that the highest prevalence of cPKU was in Turkey at 0.067%, and the lowest was in the UAE at 0.005%. Only some countries in the MENA region have established comprehensive NBS programs, such as Qatar, Saudi Arabia, United Arab Emirates, and Turkey [14], while other reviews assessing the status of NBS programs worldwide have also showed that only some countries in the Middle East, such as Bahrain, Kuwait, and Egypt, have extensive NBS programs [22,23].

### 4.1. Dietary Management of PKU

Our results revealed that 83% of patients were committed to dietary treatment (17% were not). Most patients who were not committed to dietary treatment had a higher frequency of complications than those patients committed to dietary treatment. These results highlight the importance of early dietary management in treating and preventing severe PKU complications. Early detection is the key to preventing irreversible intellectual disability and ensures optimal treatment. Diagnosis should be determined ideally between 24 and 72 h after birth to ensure an early start to treatment [24]. The diet should also be sufficient in all essential nutrients and meet each patient’s daily energy requirements to ensure their normal growth and optimal nutritional status [25]. A previous study that assessed the nutritional status of PKU patients in Jordan showed that about 50% of patients had poor physical growth and were underweight, especially during the first years of life; this may be due to a low dietary intake of protein, energy, and nutrients [26].

We have only one specialized clinic in Jordan to monitor and treat PKU patients. The clinic manages individuals with PKU from diagnosis through newborn screening into adulthood. We have a team approach in providing care to our patients. The PKU clinic team includes two pediatricians, a nurse, and three volunteer dietitians. Since the main PKU treatment is a lifelong low-protein diet, the MOH distributes only phenyl-free milk formula and low-protein flour free of charge to all age groups and nationalities throughout their lives.

Nutritional monitoring includes recording anthropometric measures (weight, height, and head circumference) to ensure normal growth. We also perform the following at each visit: food recalls, calculation of daily energy needs (daily calories), calculation of daily phenyl-free protein and natural protein requirements, provision of alternative low-phenyl recipes and meal plans and providing solutions to render phenyl-free milk more palatable. We aim to promote self-care by providing nutritional counseling and education to parents and families, particularly by teaching them how to calculate the phenylalanine content in different food products.

### 4.2. Oral Health and PKU

One of the main challenges facing PKU treatment is the high intake of simple carbohydrates as part of the treatment protocol, which may negatively impact oral health, as a high sugar intake leads to dental caries. The dietary management of PKU involves the intake of Phe-free protein substitutes, which are acidic, sweetened, and administered at least three times a day. Very few studies document the prevalence of dental caries within PKU patients [27]; however, from our clinical experience, we have observed that most of our patients do not visit a dentist and usually do not pay attention to oral hygiene. A possible reason for this is that most of our patients are from low-income families who cannot afford dentist visits regularly. Another reason is a lack of knowledge about the side effects of a high-carbohydrate diet on teeth.

Several preventative behaviors should be promoted among PKU patients to avoid dental caries. Firstly, a good dental health routine should be encouraged, and using toothpaste free from aspartame. After drinking the protein substitute, patients must drink water or rinse their mouth with water. Preferably, sugary drinks and juices should be substituted with water. Regular dental check-ups should be encouraged if the family can afford them.

Limitations of the study: One limitation of our study is that we could not collect information about an intelligence quotient (IQ) assessment, did not perform magnetic resonance imaging (MRI), or neurodevelopmental assessment due to the limited number of staff working at the PKU clinic.

### 4.3. Challenges for Jordan

One of the most significant limitations we face in Jordan is the lack of national guidelines regarding PKU treatment. We rely on up-to-date publications, international guidelines, and protocols to treat our patients; however, different information sources are not always consistent on important topics, such as the Phe levels in different fruits and vegetables, target blood Phe levels in different age groups, and the frequency of Phe testing that is necessary. Another limitation is the lack of manpower, resources, and equipment. Another drawback is the absence of metabolic dieticians, whose roles are vital in treating PKU patients. As previously mentioned, we only have one metabolic clinic in the whole country, located in Amman, which is not convenient for most of our patients living in different governorates. Living far from the health center is associated with fewer visits and less Phe testing. In addition, there is only one central laboratory specializing in Phe level testing for all our patients, resulting in a significant load on the staff and late Phe level results. The latter leads to late intervention for adjusting the Phe intake, which is especially critical in the first year of life to ensure normal brain development. Furthermore, we do not have an amino acid analyzer, which is vital in confirming PKU cases.

Lack of treatment options is one of the main challenges we face in Jordan. Due to their high cost, BH4 supplements are not available for treating PKU patients with BH4 deficiency. Special low-protein foods (SLPF) are also not available. The MOH only supports our patients with Phe-free milk formula and low-Phe flour. These issues further decrease the adherence to a low-Phe diet, which is challenging to maintain. SLPF are not available in our country, and even if a few products were available on the market, they would most likely be unaffordable to most of our PKU patients from low-income families. The availability and the cost of SLPF are important issues that the MOH must address. We do not have decent IQ and neurodevelopmental assessments or rehabilitation programs for assessment and follow-up protocols.

Although the MOH has allocated around USD 500,000 annually for PKU treatment, mainly restricted for procuring low-Phe milk and flour, there is still no envisaged funding nor a budget line allocated for other operational costs such as SLPF, supporting staff, advocacy, assessment, and monitoring. Despite the limitations encountered in Jordan, we strive to provide patients with the best services. During the COVID-19 lockdown, the genetic department at the MOH distributed Phe-free protein substitutes and flour to all of our PKU patients’ houses country-wide to avoid any patient running out of their only protein substitute.

## 5. Conclusions

The prevalence of PKU is high in Jordan, which is attributed to the high rate of consanguineous marriages. Our study highlights the importance of the NBS program in the early identification and diagnosis of new PKU cases and stresses the need for early dietary management and follow-up efforts in treatment, thereby preventing severe PKU complications. The screening program should be expanded to include more conditions.

## 6. Policy Recommendations

- Educate parents, health care providers and policy makers about NBS.
- Develop a national registry for rare diseases.
- Increase the manpower, resources, and equipment essential to the diagnosis, treatment, and follow-up of PKU patients.
- Include more specialists from the field of medical genetics, such as metabolic physicians and clinics, genetic counselors, and metabolic dieticians to assist in the treatment and dietary management of PKU patients
- Establish a laboratory specialized in measuring the PHE levels with an amino acid analyzer for confirming PKU cases and diagnosing BH4 deficiency in patients’.
- Provide treatment options and keep special low-protein foods (SLPF) at affordable prices for patients and their families.
- Provide regular intelligence quotient (IQ) assessments, magnetic resonance imaging (MRI), neurodevelopmental assessment, and rehabilitation programs.
- Allocate more financial resources and funding to PKU treatment and other operational costs such as SLPF, supporting staff, advocacy, assessment, and monitoring.

## Figures and Tables

**Table 1:** Characteristics and clinical manifestations of PKU patients.

Category	Patients (Percent) *n* = 294
Sex	
Male	169 (57.5%)
Female	125 (42.5%)
Nationality	
Jordan	267 (90.8%)
Syria	11 (3.7%)
Iraq	1 (0,3%)
Palestine	11 (3.7%)
Egypt	2 (0.7%)
Yemen	2 (0.7%)
History of consanguineous marriage	
First-degree	218 (74.2%)
Second-degree	29 (9.9%)
Third-degree	10 (3.4%)
Not consanguineous	37 (12.6%)
Discovered by screening	
Yes	165 (56%)
No	129 (44%)
Age at diagnosis	
0–7 days	28 (9.5%)
8–30 days	72 (24.5%)
More than 30 days	194 (66%)
Developmental milestone	
Delay	125 (42.5%)
No delay	169 (57.5%)
Follow-up dietary treatment	
Committed	244 (83%)
Not committed	50 (17%)
PKU phenotypes (Phenylalanine levels)	
Classic	126 (42.9%)
Mild	23 (7.8%)
Moderate	137 (46.6%)
Variant-BH 4	8 (2.7%)
Complications	
ADHD	10 (3.4%)
Mental retardation	91 (31%)
Cerebral palsy	7 (2.4%)
Epilepsy	16 ( 5.4%)
Osteopenia	1 (0.3%)
No complication	163 (55.4%)
Died	6 (2%)

**Table 2:** Effect of dietary management in prevention of PKU complications.

Follow-Up Dietary Treatment	Complications
Committed 244	84 (34%)
Not committed 50	38 (83%)

**Table 3:** Number and percentage of PKU cases identified since the 2008 establishment of the Newborn Screening Program (NBS) in Jordan.

Total Number of PKU Patients 294	Patients Born before Establishment of NBS (2008) 120 (41%)	Patients Born after Establishment of NBS (2008) 171 (58%)	*p*-Value
Mental retardation	69 (58%)	21 (12%)	0.0000
Developmental delay	86 (72%)	37 (22%)	0.0000
Dietary treatment	78 (65%)	162 (95%)	0.0000

**Table 4:** Effect of age of diagnosis on the incidence of complications from PKU.

Age at Diagnosis (Day)	Number of Patients	Complications	Mental Retardation	Developmental Delay	Cerebral Palsy
0–7	28	2 (7%)	1 (3.6%)	2 (7%)	0
8–30	72	8 (11%)	2 (2.8%)	6 (8%)	3 (4%)
More than 30	194	115 (59%)	88 (45%)	117 (60%)	4 (2%)

## Data Availability

There is no links to publicly archived datasets because of confidentiality.
